# Frequency of Discrimination, Harassment, and Violence in Lesbian, Gay Men, and Bisexual in Italy

**DOI:** 10.1371/journal.pone.0074446

**Published:** 2013-08-22

**Authors:** Concetta P. Pelullo, Gabriella Di Giuseppe, Italo F. Angelillo

**Affiliations:** Department of Experimental Medicine, Second University of Naples, Naples, Italy; Rollins School of Public Health, Emory University, United States of America

## Abstract

**Background:**

This cross-sectional study assessed the frequency of discrimination, harassment, and violence and the associated factors among a random sample of 1000 lesbian, gay men, and bisexual women and men recruited from randomly selected public venues in Italy.

**Methods:**

A face-to-face interview sought information about: socio-demographics, frequency of discrimination, verbal harassment, and physical and sexual violence because of their sexual orientation, and their fear of suffering each types of victimization.

**Results:**

In the whole sample, 28.3% and 11.9% self-reported at least one episode of victimization because of the sexual orientation in their lifetime and in the last year. Those unmarried, compared to the others, and with a college degree or higher, compared to less educated respondents, were more likely to have experienced an episode of victimization in their lifetime. Lesbians, compared to bisexual, had almost twice the odds of experiencing an episode of victimization. The most commonly reported experiences across the lifetime were verbal harassment, discrimination, and physical or sexual violence. Among those who had experienced one episode of victimization in their lifetime, 42.1% self-reported one episode in the last year. Perceived fear of suffering violence because of their sexual orientation, measured on a 10-point Likert scale with a higher score indicative of greater fear, ranges from 5.7 for verbal harassment to 6.4 for discrimination. Participants were more likely to have fear of suffering victimization because of their sexual orientation if they were female (compared to male), lesbian and gay men (compared to bisexual women and men), unmarried (compared to the others), and if they have already suffered an episode of victimization (compared to those who have not suffered an episode).

**Conclusions:**

The study provides important insights into the violence experiences of lesbian, gay men, and bisexual women and men and the results may serve for improving policy initiatives to reduce such episodes.

## Introduction

Violence is a serious public health problem worldwide either as interpersonal or collective acts [1] and it is well documented that it has immediate health effects, such as injuries and death from physical and sexual assault, psychological distress [2], anxiety [3], eating disorders [4], suicide [5], and substance abuse [6].

Among the groups at higher risk of violence and discrimination, lesbian, gay men, and bisexual may frequently be victims of prejudice, physical or sexual violence, verbal harassment, discrimination, and homophobia because of their sexual orientation [7]. Such episodes may occur in the workplace [8], in school [9,10], in forms of intimate partner violence [11,12], and in access to health care services [13,14]. These experiences of discrimination on the basis of sexual orientation, may directly contribute to a poorer health status. It is important to improve health and safety, to reduce diseases transmission and progression, and to increase mental and physical well-being by implementing antibullying policies and by providing supportive social services [15]. Relatively scarce information is available regarding the prevalence of discrimination, violence, and abuse victimization in lesbian, gay men, and bisexual [16–19] and fewer have focused on fear of victimization [20–22]. According to previous research, mainly in the United States, lifetime prevalence of any type of discrimination among this population varies from 13.1% [19] to 67.6% [23]. These results indicate that violence is widespread, although cross-study comparisons are problematic. Understanding the magnitude of the phenomenon against these groups and the characteristics of those who experience discrimination because of their sexual orientation, as possible facilitating factors that can affect the risk of suffering violence situations, are the first steps in the public health approach. Therefore, this cross-sectional study extends the literature by determining the prevalence of discrimination, verbal harassment, and physical and sexual violence and the fear of suffering violence because of their sexual orientation among a large sample of lesbian, gay men, and bisexual women and men in Italy. This study additionally explores if several variables, mainly related to socioeconomic factors, also had an effect on the different episodes of violence against this group by analyzing detailed self-reported information.

## Materials and Methods

This investigation was conducted from March to June 2011 in the city of Naples, Italy, and the study population was a systematic random sample of 1000 lesbian, gay men, and bisexual women and men recruited from nineteen randomly selected public venues such as bars, pubs, and clubs. The detailed description of the study design and methods has been published elsewhere [24]. Briefly, lesbian, gay men, and bisexual women and men previously well-trained interviewers were stationed at the entrance of the public venues and upon entering, potential participants were approached and asked to be interviewed. Participants were informed that all information gathered would be confidential, analyzed as aggregates, and no information was obtained that could lead to their identification. Participation was voluntary and those who chose to participate gave their informed consent by responding to the questions. Training was given in sampling, interview technique, and ethical issues, emphasizing the importance of safety of the participants and interviewers, minimization of under-reporting and maintaining confidentiality.

The face-to-face interview collected the following data items: socio-demographics, frequency of discrimination, verbal harassment, and physical and sexual violence because of their sexual orientation, and their fear of suffering each type of victimization. Respondents were asked to indicate number of experiences, during lifetime and in the last year, of discrimination (defined as “any act that is unfair treatment based on personal characteristics or group membership; some examples: dismissal, mobbing, exclusion, derision, unequal treatment, underestimation”); verbal harassment (defined as “any verbal conduct demonstrating hostility toward a person”); physical violence (defined as “the intentional use of physical force with the potential for causing death, disability, injury, or harm; some examples: scratching, pushing, shoving, throwing, grabbing, biting, choking, shaking, slapping, punching”); and sexual violence (defined as “any sexual act that is perpetrated against someone’s will; some examples: completed nonconsensual sex act, an attempted nonconsensual sex act, abusive sexual contact and non-contact sexual abuse”). If the interviewed respond that he/she has suffered at least one type of victimization because of his/her sexual orientation, he/she was then asked to describe the last three experiences suffered and for each of them, the type and the place where the episode occurred, the perpetrator, and her/his/their sexual orientation, injuries reported, whether he/she had asked someone for help after the aggression, and whether, in a 10-point Likert scale with options ranging from 1 (not at all) to 10 (very much), he/she considered serious the experiences suffered.

For each type of victimization, respondents were requested to report their own perceived fear of suffering discrimination, verbal harassment, and physical or sexual violence because of their sexual orientation. The perceived fear measures was based on 10-point Likert scale with the lower score labeled as 1 = not at all fear and the higher score as 10 = very much fear. Higher score was indicative of greater fear.

The questionnaire developed was pilot tested with 50 lesbian, gay men, and bisexual to check the content, readability, and comprehensiveness of items. A group of experts reviewed the format and content of the items, as well as the content validity of the instrument as a whole. The internal reliability was assessed using Cronbach’s α.

Ethics Committee approval was obtained from the Second University of Naples before the study initiation.

### Statistical analysis

Univariate and multivariate analyses were performed to assess the effect of the independent variables on the different outcomes of interest. The chi-square test or Fisher’s exact test was used to explore the statistical differences between categorical variables. The independent samples *t*-test was used to compare statistical difference between continuous variables in two groups. Then, logistic regression has been performed to explore the impact of the variables on dichotomous outcomes of interest. Two multivariate logistic regression models were constructed: a) have suffered at least one type of victimization because of their sexual orientation (no = 0; yes = 1) (Model 1); fear of suffering any kind of violence because of their sexual orientation (no = 0; yes = 1) (Model 2). For the purposes of analysis, in Model 2, a dichotomous variable was created that compared respondents with a fear of suffering any kind of violence because of their sexual orientation with a value from 1 to 5 to respondents with a value from 6 to 10. The following variables irrespective of the results of the univariate analysis were included into both models: gender (male=0; female=1), age (continuous, in years), educational level (three categories: middle school or lower=1; high school = 2; college degree or higher=3), sexual orientation (three categories: lesbian=1; gay men=2; bisexual=3), occupation (unemployed=0; employed=1), and marital status (not married=0; other = 1). The variable number of other persons in the household (anyone = 0; more than one=1) was also included in Model 1; the variables have suffered at least one type of victimization because of their sexual orientation (no = 0; yes = 1) and being a member of a homosexual association (no = 0; yes = 1) were also included in Model 2. Backward stepwise selection procedures were applied so that the final multivariate models only included variables providing a significant explanation of outcomes, a significance level of 0.2 was used as limit for variables to enter in the model and 0.4 for variables to retain. Adjusted odds ratios (ORs) as well as their 95% confidence intervals (CIs) were calculated performing the logistic regression analyses. All statistical tests were two-tailed and the results were considered statistically significant at a *p*-value of less than 0.05. Stata 10.1 software package [25] was used for performing all statistical analyses.

## Results

The results of the pilot study showed a good internal consistency with a Cronbach’s α over 0.9.

The response rate was 86.8% and the data regarding socio-demographic characteristics were reported elsewhere [24]; briefly of the 1000 respondents, almost two-thirds were male, the mean age was 26.6±6.8 years (range 25-81), 56.6% (n=566) were gay men, 27.9% (n=279) were lesbian, and 15.5% (n=155) were bisexual women and men.

Table 1 presents the prevalence of the different types of victimization in the study population occurred in their lifetime and in the last year because of their sexual orientation according to several characteristics and the significance levels of univariate analysis to examine the association of these characteristics on receiving the various forms of victimization. A total of 283 (28.3%) participants self-reported that they had experienced at least one episode of victimization in their lifetime. Among those respondents who have reported such experience across the lifetime, the most commonly reported experience was verbal harassment (85.2%), whereas lower frequencies were indicated for discrimination (28.6%), and physical violence or sexual violence (26.2%). A total of 11.9% among the whole sample and of 42.1% among those who had experienced at least one episode of victimization in their lifetime because of the sexual orientation self-reported at least an episode of victimization in the past year. Among those respondents who have reported such experience in the past year respectively, 84%, 21.9%, and 15.1% had reported verbal harassment, discrimination experience, and physical violence or sexual violence.

**Table 1:** Prevalence of the different types of victimization occurred in lifetime and in the last year in the study population.

	**Experienced at least one episode of victimization**				**Verbal harassment**				**Discrimination**				**Physical or sexual violence**			
	**Lifetime**		**In the last year**		**Lifetime**		**In the last year**		**Lifetime**		**In the last year**		**Lifetime**		**In the last year**	
	**n=283**	**28.3%**	**n=119**	**42.1%**	**n=241**	**85.2%**	**n=100**	**84%**	**n=81**	**28.6%**	**n=26**	**21.9%**	**n=74**	**26.2%**	**n=18**	**15.1%**
***Characteristic***	**n**	**%**	**n**	**%**	**n**	**%**	**n**	**%**	**n**	**%**	**n**	**%**	**n**	**%**	**n**	**%**
*Gender*																
Male	166	58.7	69	58	145	60.2	58	58	51	63	15	57.7	44	59.5	15	83.3
Female	117	41.3	50	42	96	39.8	42	42	30	37	11	42.3	30	40.5	3	16.7
	0.07ˇ		0.84ˇ		0.22ˇ		1^*^		0.35ˇ		0.89ˇ		0.87ˇ		0.02*	
*Age (years)*	26.9±6.7 (17-64)^+^		26±6 (17-43)^+^		26.3±5.9 (17-45)^+^		25.9±5.9 (17-42)^+^		27.5±7.2 (17-62)^+^		26.1±6.5 (18-43)^+^		27±7.3 (17-64)^+^		24.5±5.9 (17-38)^+^	
	0.36˜		0.05˜		0.0003˜		0.44˜		0.35˜		0.51˜		0.84˜		0.26˜	
*Sexual orientation*																
Gay men	154	54.4	62	52.1	135	56	52	52	49	60.5	14	53.9	39	52.7	13	72.2
Lesbian	92	32.5	34	28.6	72	29.9	27	27	23	28.4	7	26.9	26	35.1	3	16.7
Bisexual	37	13.1	23	19.3	34	14.1	21	21	9	11.1	5	19.2	9	12.2	2	11.1
	0.09ˇ		0.03ˇ		0.06ˇ		1^*^		0.43ˇ		0.74^*^		0.85ˇ		0.22*ˇ	
*Marital status*																
Not married	257	90.8	109	91.6	220	91.3	91	91	74	91.4	22	84.6	65	87.8	17	94.4
Other	26	9.2	10	8.4	21	8.7	9	9	7	8.6	4	15.4	9	12.2	1	5.6
	0.002ˇ		0.7ˇ		0.56^*^		1^*^		0.84ˇ		0.28^*^		0.3ˇ		1^*^	
*Educational level*																
Middle school or lower	44	15.6	22	18.5	37	15.4	19	19	11	13.6	5	19.2	13	17.6	4	22.2
High school	161	57.1	64	53.8	136	56.7	53	53	42	51.8	11	42.3	51	68.9	12	66.7
College degree or higher	77	27.3	64	27.7	67	27.9	28	28	28	34.6	10	38.5	10	13.5	2	11.1
	<0.001ˇ		0.47ˇ		0.86ˇ		1^*^		0.22ˇ		1^*^		0.008ˇ		1^*^	
*Occupation*																
Employed	250	88.3	109	8.4	215	89.2	92	92	70	86.4	23	88.5	62	83.8	16	88.9
Unemployed	33	11.7	10	91.6	26	10.8	8	8	11	13.6	3	11.5	12	16.2	2	11.1
	0.54ˇ		0.15ˇ		0.27ˇ		1^*^		1^*^		0.53^*^		0.16ˇ		0.65^*^	
*Openness with others regarding sexual orientation*																
Anyone	3	1.1	3	2.5	3	1.2	3	3	-	-	-	-	1	1.4	1	5.6
Others	280	98.9	116	97.5	238	98.8	97	97	81	100	26	100	73	98.6	17	94.4
	0.11^*^		0.07^*^		1^*^		1^*^		-		-		1^*^		0.39^*^	
*Number of other persons in the household*																
Anyone	19	6.7	9	7.6	15	6.2	8	8	9	11.1	4	15.4	6	8.1	1	5.6
More than one	264	93.3	110	92.4	226	93.8	92	92	72	88.9	22	84.6	68	91.9	17	94.4
	0.12ˇ		0.63^*^		0.5^*^		1^*^		0.06ˇ		1^*^		0.58ˇ		1^*^	

Numbers for each item may not add up to total number of study population due to missing values

^+^ Mean±Standard deviation (Range)

ˇChi squared *p*-value; ˜*t*-test *p*-value; ^*^ Fisher’s exact test *p*-value

The results of the univariate statistical analysis showed that, among demographic and socioeconomic characteristics, only the marital status (*p*=0.002) and the educational level (*p*<0.001) were significantly associated with victimization occurred in the lifetime because of the sexual orientation. Following univariate analysis, a multivariate logistic regression analysis was conducted to evaluate the variables as predictors with the experience of at least an episode of victimization in the lifetime because of the sexual orientation as the dependent variable. The results of the final model are presented in Table 2. The results substantially confirmed the findings of the first step of the analysis. Indeed, those unmarried, compared to the others, were more likely to have experienced an episode of victimization (OR=0.49; 95% CI=0.31-0.77). Compared to bisexual individuals, lesbian respondents had almost twice the odds of experiencing an episode of victimization (OR=1.68; 95% CI=1.07-2.65). The education has also impact on having suffered at least one type of violence since respondents with high school (OR=0.53; 95% CI=0.39-0.71) were less victim compared to those with a college degree or higher.

**Table 2:** Multivariate logistic regression analysis to characterize factors associated with the different outcomes of interest.

Variable	OR	SE	95% CI	*p* value
**Model 1**. Have suffered at least one type of violence because of their sexual orientation				
Log likelihood=-576.25, χ^2^=35.85 (5 df), *p*<0.0001				
Educational level				
High school	0.53	0.08	0.39-0.71	<0.001
College degree or higher*	1.0	-	-	-
Who is not married	0.49	0.11	0.31-0.77	0.002
Who live alone	0.67	0.21	0.37-1.22	0.19
Sexual orientation				
Bisexual*	1.0	-	-	-
Lesbian	1.68	0.39	1.07-2.65	0.026
Gay men	1.22	0.27	0.8-1.87	0.36
**Model 2**. Have fear of suffering any kind of violence because of their sexual orientation				
Log likelihood=-623.1, χ^2^=84.77 (7 df), *p*<0.0001				
Who have suffered at least one type of violence because of their sexual orientation	2.06	0.31	1.54-2.76	<0.001
Sexual orientation				
Bisexual*	1.0	-	-	-
Lesbian	2.5	0.65	1.5-4.2	<0.001
Gay men	2.3	0.81	1.16-4.58	0.018
Who is not married	0.61	0.13	0.4-0.91	0.016
Women	2.23	0.91	1.01-4.94	0.049
Educational level				
Middle school or lower	1.37	0.29	0.91-2.1	0.14
College degree or higher*	1.0	-	-	-
Who is a member of a homosexual association	0.86	0.14	0.62-1.19	0.35

^*^ Reference category

For each type of victimization, perceived fear of suffering a type of violence, measured on a 10-point Likert scale with a lower score indicating a lower fear, ranges from a value of 5.7±2.8 for verbal harassment to a value of 6.4±2.5 for discrimination. Only 38.6% reported fear of suffering any type of victimization because of their sexual orientation. The logistic regression model showed that participants were more likely to have fear of suffering any type of victimization because of their sexual orientation if they were female compared to male (OR=2.23; 95% CI=1.01-4.94) and unmarried compared to the others (OR=0.61; 95% CI=0.4-0.91). Respondents who have already suffered at least one type of victimization were more likely to have fear than those who have not suffered an episode (OR=2.06; 95% CI=1.54-2.76). Moreover, when bisexual was chosen as reference category, a value of odds of 2.5 was detected for lesbian (95% CI=1.5-4.2) and 2.3 for gay men (95% CI=1.16-4.58).

## Discussion

To our knowledge the present study is the most extensive investigation regarding violence against a sample of lesbian, gay men, and bisexual women and men conducted in Italy, and the identification of the various risk factors is an important step towards developing efficacious interventions for reducing violence against this population. Public health practitioners have been advocating for more focused attention in meeting their needs. Violence among lesbian, gay men, and bisexual does not receive the same attention as it does among the heterosexual group despite the fact that in this population occurs at similar or higher rates as in heterosexual group. A challenge lies in reaching those who experience violence, and ensuring that those who seek help are effectively served.

In the present study, 28.3% of the participants reported that they had experienced at least one episode of victimization because of their sexual orientation in their lifetime and 42.1% of them revealed a discrimination in the last year. The lifetime prevalence was particularly low in comparison with that depicted from the United States, with values ranging from 50.8% in homosexual or bisexual men [26] to 67.6% in lesbian, gay, and bisexual [23]. Moreover, in the United States 13.1% of lesbians, bisexuals, and gay men had experienced violence during their adult life [19] and more than one third of gay, lesbian, and bisexual employees had experienced sexual orientation discrimination [21]. The prevalence of any type of discrimination in the last year of 11.9% among the whole sample in the present study, was lower than the 21.4% [27] and 61.3% [23] among lesbian, gay, and bisexual and the 60% and 58% among men who have sex with men [28]. Finally, values ranged from 5.6% for physical assault to 37.4% for discrimination for gay/lesbian, and from 8.9% for physical assault to 32.6% for verbal threat of harm for bisexual [29]. The difference observed with the findings from previous experiences is highly relevant and this may partially be explained by the nature of the population, the sampling and recruitment strategies, the cultural attitudes, and the decade in which the studies were conducted.

Using exploratory multivariate logistic regression analysis, a few factors were associated with the two outcomes of interest. Of the several socio-demographic characteristics analyzed, gender, marital status, and level of education were the significant important predictors. Specifically, females and those unmarried were most likely to have experienced at least an episode of victimization in their lifetime because of the sexual orientation and to have higher levels of perceived risk and fear of victimization. The result regarding the gender is in line with another study [20]. Moreover, lower educational level was found to be associated with the experience of victimization and this may be partly explained by the fact that those more educated possess the skills necessary to better recognize a potentially dangerous situation. Educational attainment has been already found to reduce the likelihood of violence [30–32]. It is interesting to note that having already experienced an episode of victimization play a role regarding the fear of suffering any type of victimization because of their sexual orientation because these participants were more likely to have a higher fear. This finding is in accordance with a previous study [20] and has important implications for the safety of such sample.

The findings should be interpreted while considering some potential limitations of the study methodology. First, a cross-sectional design has been used and, consequently, as with all cross-sectional research, it is not possible to establish the direction of the associations between violence experience and the different variables studied and, therefore, there are uncertainties about the temporal relationships between the outcomes measured and the study variables. Although the interaction of victimization experiences and several variables has been examined, this investigation was not designed to explain this interaction fully. Second, possible biases, like any study based on gathering information through face-to-face interview, affecting the frequency of individual victimization experiences may have resulted from the method used. This method, while enabling full response-rate on all variables, might have determined either under-reported or exaggerated the actual amount of these experiences. However, there were reasons to believe that the data are accurate, as the collection procedure was performed with great care by lesbian, gay men, and bisexual women and men interviewers and this may possibly have contributed to the creation of trust and confidence between the interviewer and the participants. This assumption is supported by the extremely low non-response rate. Despite these limitations, strengths of the study include a careful pre-testing of the questionnaire, rigorous interviewer training, the emphasis on ethical considerations, the high response rate, and that the participants were assured that their responses would remain anonymous. This study contributes to establish the extent of violence experiences in lesbian, gay men, and bisexual women and men in Italy for the first time.

In conclusion, this study provides important insights into the violence experiences of lesbian, gay men, and bisexual women and men and the results may serve as an effective focal point for improving policy community-based support.
